# Two new species of the genus *Aphis* (Hemiptera, Aphididae) from Chile on host species of Alstroemeriaceae and Ericaceae

**DOI:** 10.3897/zookeys.738.21966

**Published:** 2018-02-19

**Authors:** Sara I. Lopez Ciruelos, Paul A. Brown, Juan M. Nieto Nafria

**Affiliations:** 1 Servicio de Microscopia; Universidad de Leon, 24071 Leon, Spain; 2 Department of Life Sciences; Natural History Museum. Cromwell Road. London, SW7 5BD, United Kingdom; 3 Departamento de Biodiversidad y Gestion Ambiental, Universidad de Leon, 24071 Leon, Spain

**Keywords:** Aphididae, aphids, Chile, *Gaultheria*, *Luzuriaga*, New species

## Abstract

Specimens from Southern Chile belonging to two undescribed aphid species held at the Natural History Museum in London have been studied. *Aphis luzuriagae* **sp. n.** collected on *Luzuriaga radicans* and on other unidentified *Luzuriaga* species (Alstroemeriaceae) is described from apterous and alate viviparous females. *Aphis gaultheriae* **sp. n.** collected on *Gaultheria mucronata* (Ericaceae) is described from apterous viviparous females. Taxonomic peculiarities of both species are discussed.

## Introduction

Specimens belonging to several aphid samples collected by D. Hille Ris Lambers during field work in Chile in 1974 on the very injurious attacks on cereal crops by *Schizaphis graminum* (Rondani, 1852), are preserved in the aphid collection of the Natural History Museum in London. Blackman and Eastop (2017) include in the keys of aphids living on *Luzuriaga* (Alstroemeriaceae) and on *Gaultheria* (Ericaceae), two unnamed species of *Aphis* collected by Hille Ris Lambers during this field work. In this paper, these two new species are described as *Aphis luzuriagae* sp. n. and *Aphis gaultheriae* sp. n.

## Material and methods

Studied specimens are mounted on microscopic slides using Canada balsam. Collection data for the studied specimens of the new species are included in the Types section for each new species. For comparison, specimens of other species recorded in South America preserved in the collection of the University of Leon have also been studied. Papers describing these species, and in particular the comparative table of features of South American *Aphis* species presented by Lopez Ciruelos et al. (2017), have been consulted. Measurements of specimens were taken as detailed by Nieto Nafria and Mier Durante (1998). The descriptions of *Aphis vurilocensis* Nieto Nafria, Brown & Lopez Ciruelos, 2017 and of *Aphis cuyana* Lopez Ciruelos & Ortego, 2017 (Gonzalez Rodriguez et al. 2017; Lopez Ciruelos et al. 2017) have been used as models for the descriptions of the new species. Data on aphid host plants and distribution of plant species have been respectively taken from Blackman and Eastop (2017) and WCSP (2017). Photographs have been taken using an Olympus set: BX61 microscope, DP70 digital camera and DP Manager Software version 2.1.1.163.

## Taxonomy

### Aphis (Aphis) luzuriagae sp. n.

Taxon classificationAnimaliaHemipteraAphididae

http://zoobank.org/C1F8F288-747A-404A-83F7-E9546F33D568

#### Types.

Holotype, apterous viviparous female (measured specimen number 5, mounted with two nymphs): CHILE, Region VII Bio Bio, province Nuble, East of Chillan “fundo Borges”, 7 November 1974, on *Luzuriaga radicans*, Hille Ris Lambers *leg.* [867], Natural History Museum collection (BMNH(E) 1984-340). Paratypes: 27 apterous viviparous females [apt] and 5 alate viviparous females [al] mounted on 20 slides, Natural History Museum collection (BMNH(E) 1984-340); same data as the holotype (4 apt); same locality and date and sample number as the holotype, on *Luzuriaga* sp. (1 apt & 1 al); same locality, date and sample number, on *Luzuriaga* sp. [*Luzuriaga falcata* on the label, a name that is unknown to us] (6 apt & 4 al); CHILE, Region IX La Araucania, province Cautin, Temuco “cerro Nielol”, 18 November 1974, on *Luzuriaga* sp., Hille Ris Lambers *leg.* [908] (1 apt); CHILE, Region X Los Lagos, province Osorno, Puyehue National Park, 21 November 1974, on *Luzuriaga radicans*, Hille Ris Lambers *leg.* [910] (2 apt); CHILE, Region X Los Lagos, province Chiloe, Huillinco lake, 23 November 1974, on *Luzuriaga* sp., Hille Ris Lambers *leg.* [918] (3 apt) and Tollenaar *leg.* [Hille Ris Lambers 919] (10 apt.).

#### Apterous viviparous females.

**Figure 1.**
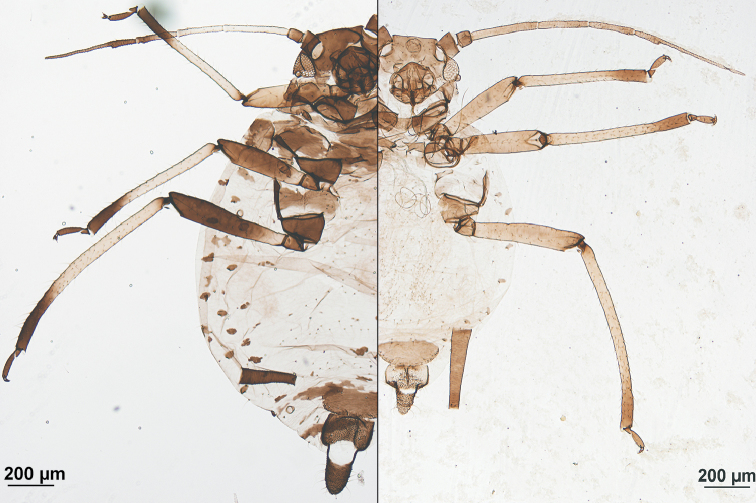
Apterous viviparous females, habitus (in part). Left, *Aphis alstroemeriae*; right, *Aphis luzuriagae* sp. n., paratype.

**Figure 2.**
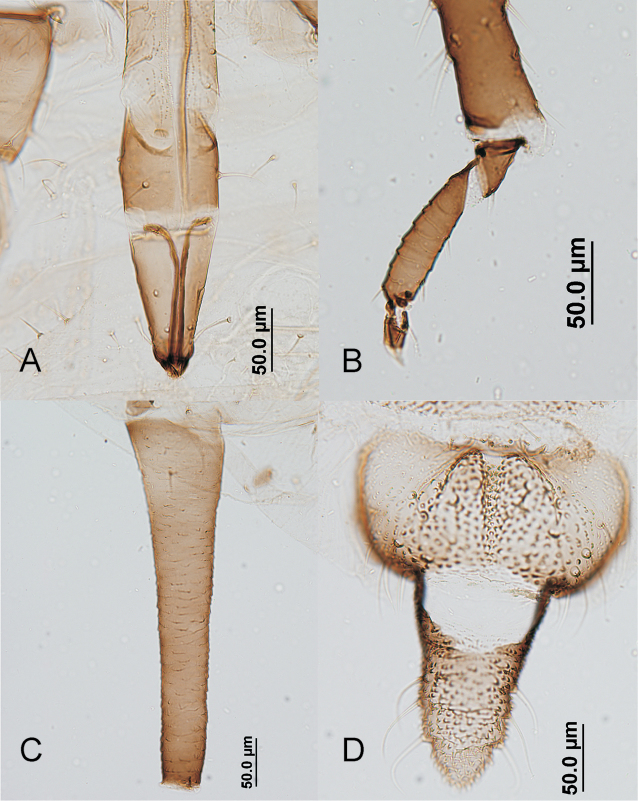
*Aphis luzuriagae* sp. n., apterous viviparous female. **A** end of rostrum **B** end of tibia and tarsus of hind leg **C** siphunculus **D** cauda and anal plate.

(Figs 1, 2), from 28 specimens, of which 25 have been measured. Colour when alive unknown. When prepared pale in general, with head, including clypeus, mandibular and maxillary lames, antennae, rostrum, legs, dorso-thoracic sclerites, spiracular sclerites, genital and anal plates, and cauda tenuously brown pigmented, and siphunculi brown to dark brown. Body 1.37–1.95 mm long, 1.76–2.52 times hind tibia (0.68–1.03 mm) and 4.09–6.29 times siphunculus. Head with a marked ventral protuberance, which is long-elliptical, rugous and carries two setae. Antenna 1.13–1.61 mm and 0.66–0.92 times body length. Antennal segment III 0.25–0.39 mm and 0.68–0.96 times segment VI processus terminalis, dorsally near smooth, and with 6–12 blunt pale setae, 12–23 µm and 0.4–0.8 times subarticular diameter of antennal segment III [*D*], shorter than those on vertex (22–30 µm and 0.8–1.1 times *D*), which are similar in shape. Antennal segments IV and V respectively 0.16–0.28 and 0.15–0.22 mm. Antennal segment VI processus terminalis 0.32–0.45 mm and 2.67–3.82 times base of segment (0.10–0.13 mm). Rostrum reaching to or slightly beyond the hind coxae; ultimate rostral segment 0.11–0.13 mm, 0.96–1.20 times base of antennal segment VI, 1.00–1.33 times second segment of hind tarsi, relatively broad with convex sides and with 2 long and fine accessory setae. Dorsum of thorax usually with wide but lightly pigmented marginal sclerites. Prothoracic marginal tubercles low and relatively wide, nearly similar in volume to triommatidium. Inside setae on hind trochanter 32–53 µm and 0.5–1.1 times trochantrofemoral suture; dorsal setae on hind femora 15–25 µm and 0.5–1.0 times *D.* Tarsi and apex of tibiae darker than the rest of legs, except the femoro-tibial joint which is nearly black. Apical brown portion of tibiae larger than rest of segment. Tarsi very short, first segments with (2)3.3.3(2) setae. Marginal tubercles on abdominal segments 1 and 7 small, their diameter is shorter than length of closest marginal setae, and sometimes lacking on some segments and/or sides. Intermediate abdominal segments without marginal tubercles. Dorsum of abdomen without sclerites other than spiracular ones, which are small and brown. Dorsal setae on thorax and abdomen also blunt or slightly pointed; marginal setae on intermediate abdominal segments 17–25 µm and 0.6–1.0 times *D.* Abdominal segment 8 with two setae, 27–35 µm and 0.8–1.6 times *D.* Siphunculi tapering in general, with apical portion slightly enlarged, poorly ornamented and without flange, 0.26–0.42 mm, 1.93–2.59 times cauda, and (4.33)5.09–6.67(7.10) times its diameter at mid length. Cauda robust, nearly triangular, the proximal part being straight-sided without or with a very small constriction, sometimes more pigmented towards apex, 0.12–0.17 mm and 1.04–1.35 times its basal width, with 4–7 long, delicate and curved setae.

#### Alate viviparous females.

From 5 specimens, all of them measured. Very similar to apterous viviparous females but with darker head, antennae and legs, antennal segment III imbricate-spinulose and brown thorax; two specimens lack dorsal abdominal pigmented sclerites, one specimen has lightly pigmented and spinulose abdominal sclerites: small marginal on segment 1, wide marginal on segments 2–6, and broad transverse on each of segments 7 and 8, and two other specimens exhibit intermediate sclerotization and pigmentation. Secondary sensoria on antennal segments III and IV; 6–10 on III extended along segment and 1–4 on IV. Body 1.50–1.80 mm long, 1.76–1.77 times hind tibia (0.85–1.00 mm) and 5.17–6.04 times siphunculus. Antenna 1.41–1.72 mm and 0.88–1.00 times body length. Antennal segment III 0.31–0.42 mm and 0.71–0.88 times segment VI processus terminalis. Setae on antennal segment III 15–18 µm and 0.5–0.8 times *D.* Setae on vertex 17–23 µm and 0.6–1.0 times *D.* Antennal segment IV 0.22–0.29 mm. Antennal segment V 0.18–0.25 mm. Antennal segment VI processus terminalis 0.43–0.47 mm and 3.58–4.33 times segment VI base (0.10–0.13 mm). Ultimate rostral segment 0.11–0.12 mm, 0.92 –1.14 times base of antennal segment VI and 1.15–1.20 times second segment of hind tarsi. Inside setae on hind trochanter 37–40 µm and approximately 0.8 times trochantrofemoral suture. Dorsal setae on hind femora 17–23 µm and 0.6–1.0 times *D.* Marginal setae on intermediate abdominal segments 20–23 µm and 0.7–1.0 times *D.* Setae on abdominal segment 8, 27–30 µm and 1.0–1.3 times *D.* Siphunculus 0.26 –0.30 mm, 2.12–2.41 times cauda, and 4.83–6.78 times its diameter at mid length. Cauda 0.11–0.13 mm and 0.96–1.14 times its basal width, with 4–7 setae.

#### Sexuales.

(Oviparous females and males). Unknown.

#### Host plant.

All collected specimens of *A. luzuriagae* sp. n. have been found on plants of several species of *Luzuriaga* (Alstroemeriaceae). *Luzuriaga* species are distributed in the South of Argentina and in Central and Southern Chile.

#### Etymology.

The specific name “*luzuriagae*” is the plant host genus name of the new aphid species, in genitive.

#### Taxonomic discussion.

Only two other genera of this plant family have been recorded hosting aphids: *Alstroemeria* and *Bomarea*, but only one species belonging to *Aphis* has been recorded: *A. alstroemeriae* Essig, 1954, on *Alstroemeria* sp. in Chile (Nieto Nafria et al. 2016). The differences between the apterae of this species and *Aphis luzuriagae* sp. n. are very conspicuous and evident (Fig. 1). The new species can be distinguished from other South American *Aphis* species by the combination of the following features: 1) in apterous viviparous females: the absence of dorsal abdominal pigmented sclerotization, even intersegmental, anteroventral cephalic protuberance, very short tarsi, small or absent marginal tubercles on abdominal segments 1 and 7, lack of marginal tubercles on intermediate abdominal segments, and relatively long processus terminalis of VI antennal segment; 2) in alate viviparous females: secondary sensoria extend over all of antennal segment III and present on antennal segment IV, and dorsal abdominal sclerotization (marginal sclerites 1–6 and transverse bands 7–8) absent, more or less insinuated or conspicuously present.

### Aphis (Aphis) gaultheriae sp. n.

Taxon classificationAnimaliaHemipteraAphididae

http://zoobank.org/BBC6836C-6829-48D8-9EFA-BD69CA76C834

#### Types.

Holotype apterous viviparous female (measured specimen number 1), CHILE, Region X Los Lagos, province Chiloe, Lake Huillinco, on *Gaultheria mucronata* (L. fil.) E. J. Remy [*Pernettya mucronata* on the label], 24 November 1974, Hille Ris Lambers *leg.* [928], mounted with two nymphs, Natural History Museum collection (BMNH(E) 1984-340). Paratypes: 2 apterous viviparous females collected with the holotype, mounted in two slides with nymphs, Natural History Museum collection (BMNH(E) 1984-340).

#### Apterous viviparous females.

**Figure 3.**
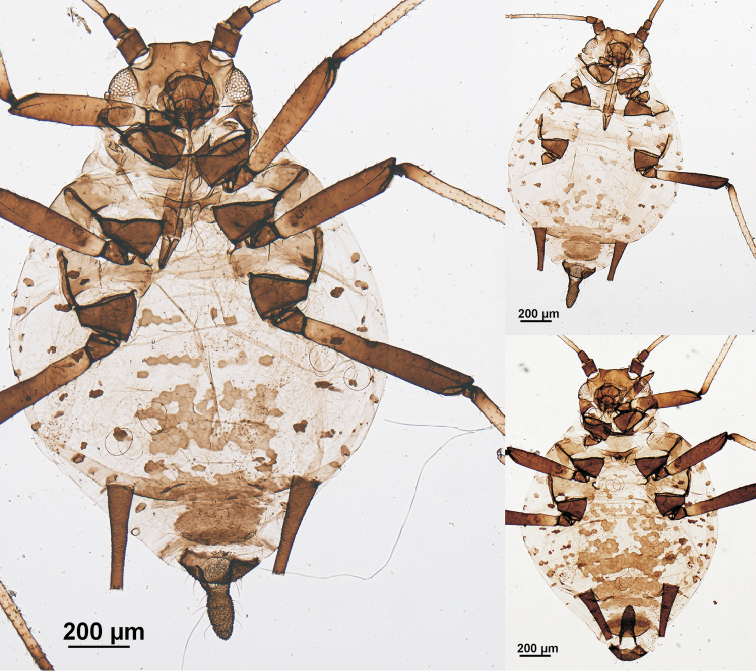
*Aphis gaultheriae* sp. n., apterous viviparous females; holotype, specimen on the left.

**Figure 4.**
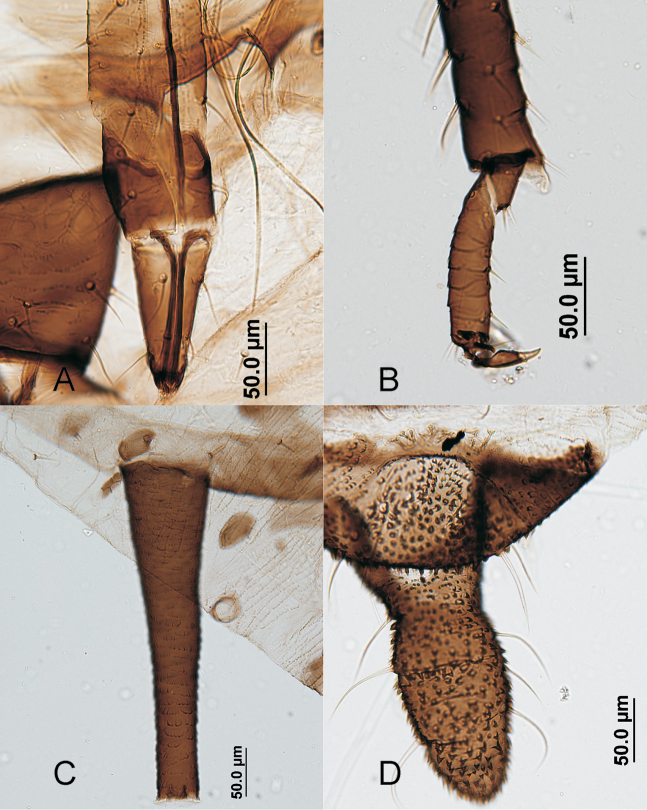
*Aphis gaultheriae* sp. n., apterous viviparous female. **A** end of rostrum **B** end of tibia and tarsus of hind leg **C** siphunculus **D** cauda.

(Figs 3, 4), from 3 specimens, all of them measured. Colour when alive unknown. When prepared, head, including clypeus, mandibular and maxillary lames brown; antennal segments I and II dark brown like segment VI; rostrum brown, coxae, trochanters, and most of femora dark brown (like antennal segment I), tibiae mainly very pale brown (like antennal segments III–V); dorsal thoracic and abdominal sclerites and genital plate brown (lighter than head), siphunculi, anal plate and cauda dark brown. Body 1.85–2.08 mm long, 1.88–2.24 times hind tibia (0.83–1.05 mm) and 5.49–6.27 times siphunculus. Antenna 1.25–1.56 mm and 0.68–0.79 times body length. Antennal segment III 0.35–0.43 mm and 1.32–1.42 times segment VI processus terminalis, dorsally almost smooth, and with 8–10 thick, pointed and very pale setae, 15–20 µm and 0.7–1.0 times subarticular diameter of antennal segment III [*D*], shorter than those on vertex (27–30 µm and 1.2–1.5 times *D*). Antennal segments IV and V respectively 0.18–0.25 and 0.20–0.26 mm. Segment VI processus terminalis 0.25–0.33 mm and 2.13–2.50 times segment VI base (0.12–0.14 mm). Rostrum slightly extending beyond the mid-coxae; ultimate rostral segment long (0.10–0.12 mm, 0.74–0.96 times base of antennal segment VI, 0.80–1.00 times second segment of hind tarsi), straight and with 2 very long and fine accessory setae. Dorsum of thorax with wide marginal sclerites and several pleural and spinal spots, paler than intersegmental sclerites and metathorax/first abdominal segment. Prothoracic marginal tubercles erect, protuberant and smaller than triommatidium. Inside setae on hind trochanter 37–40 µm and 0.67–0.73 times trochantrofemoral suture; dorsal setae on hind femora 25 µm and 1.1–1.3 times *D.* First segment of tarsi with 3 setae. Protuberant marginal tubercles on abdominal segments 1 and 7 delicate, but only those on segment 1 are especially delicate. Intermediate abdominal segments without marginal tubercles. Intersegmental sclerites darker than segmental and spiracular sclerites. Abdominal segments 1–4 with more or less abundant polygonal spinal sclerites that can be coalescent, and usually without marginal sclerites (polygonal cells are present in one specimen); abdominal segments 5 and 6 each with wide transverse spinopleural band, and abdominal segments 7 and 8 with individual narrow transverse stripes; this variability is similar to that presented by *A. tehuelchis* Nieto Nafria & Lopez Ciruelos, 2016 (Lopez Ciruelos et al. 2016). Dorsal setae on thorax and abdomen also thick and pointed; marginal setae on intermediate abdominal segments 17–25 µm and 0.9–1.3 times *D.* Abdominal segment 8 with 2 setae, 20–22 µm and 0.9–1.1 times *D.* Siphunculi tapering on proximal 1/2–2/3 and distally cylindrical, with small flange and ornamentation of spinuled scales, 0.30–0.36 mm, 1.11–1.38 times cauda, and 6.00–6.56 times its diameter at mid length. Cauda robust finger-shaped, 0.26–0.27 mm and 1.93–2.30 times its basal width, with 10–11 long, delicate and curved setae.

#### Alate viviparous females and sexuales.

(Oviparous females and males). Unknown.

#### Host plant.

The type specimens of *Aphis gaultheriae* sp. n. were collected on *Gaultheria mucronata* (Ericaeae), which is known from Peru, Bolivia and southern Argentina and Chile (and introduced in Britain and Ireland).

#### Etymology.

The specific name “*gaultheriae*” is the plant host genus name of the aphid, in genitive.

#### Taxonomic discussion.

Species of 33 genera of Ericaceae have been reported as host plants of aphid species around the World, but only 16 host species of *Aphis*, many of which are polyphagous. Only seven *Aphis* species specializing on plant species belonging to Ericaceae are known: *A. arbuti* Ferrari, 1872, *A. callunae* Theobald, 1915, *A. madronae* Essig, 1926, *A. multiflorae* Barbagallo & Stroyan, 1982, *A. remaudieri* Borner, 1952, *A. uvaeursi* Ossiannilsson, 1959 and *A. vaccini* (Borner, 1914), which are European in distribution. Apterous viviparous females of *Aphis gaultheriae* sp. n. are recognizable by having spinal abdominal sclerotization, present at least on segments 5 to 8, extensively black femora, broad cauda more or less pigmented like siphunculi and similar to that of *A. spiraecola*, and an ultimate rostral segment no longer than the second segment of the hind tarsi.

## Supplementary Material

XML Treatment for Aphis (Aphis) luzuriagae

XML Treatment for Aphis (Aphis) gaultheriae
